# Research on Knowledge Learning of COVID-19 Video Viewers: Based on Cognitive Mediation Model

**DOI:** 10.3390/healthcare11040570

**Published:** 2023-02-14

**Authors:** Jingfang Liu, Caiying Lu, Jingxian Cai

**Affiliations:** School of Management, Shanghai University, No. 20, Chengzhong Road, Jiading District, Shanghai 201899, China

**Keywords:** cognitive mediation model, dual coding theory, COVID-19 videos, knowledge learning

## Abstract

During the COVID-19 epidemic, social media has become the main channel for people to learn information related to the epidemic, among which information in the form of videos has played a significant role in the prevention and control of COVID-19. However, few studies have analyzed the process of knowledge learning of individuals through watching COVID-19 videos. Therefore, to explore the process of COVID-19 video viewers’ knowledge acquisition, this paper constructs a knowledge learning path model based on the cognitive mediation model and dual coding theory. A sample of 255 valid questionnaires was collected to validate this model. The results of this study show that an individual’s perceived risk of COVID-19 affects their surveillance motivation positively, while surveillance motivation further stimulates the attention and elaboration about the information in COVID-19 videos. Among them, attention positively influences the elaboration about the information. Ultimately, both an individual’s attention and elaboration positively influence the knowledge he or she acquires from the COVID-19 videos. This paper not only verifies the hypothesized relationships in the original cognitive mediation model, but also extends the model to the context of video knowledge learning. Analyzing the knowledge learning process of COVID-19 video viewers, this paper can provide suggestions for government propaganda departments and relevant media to improve public knowledge of COVID-19.

## 1. Introduction

At the end of 2019, a sudden new coronavirus outbreak shattered the balance of the world. A new virus, the 2019 New Coronavirus, has swept the world with its rapid spread and devastating hazards, causing widespread public panic and dealing a major blow to the stability of society [1]. As the epidemic continues to grow, more than 500 million cases of infection and over 6 million deaths were reported as of June 2022. The epidemic has mercilessly claimed countless living lives, disrupted the rhythm of people’s lives, and caused great trauma to their physical and mental health. During the epidemic, social media became an important channel for the public to get the latest news about COVID-19 and learn about preventive measures [2]. People’s restricted travel and increased time spent at home led to a surge in social media use behavior. As a result, people rely more heavily on social media for information related to COVID-19, to relieve psychological anxiety, and to prevent COVID-19 [3]. The popularity of short videos at the moment has made video-based information content popular with the public. Along with the development of the epidemic, a large amount of video content related to COVID-19 has been created and has received a lot of attention and viewing. The COVID-19 videos have played a major role in containing the epidemic and reducing anxiety by disseminating informative content related to the COVID-19 outbreak [4]. Therefore, COVID-19 videos are often used in the prevention and control of epidemics [5].

This epidemic has lasted for three years, and the prevention and control of the epidemic is also a constant battle. As the epidemic battle lengthens, the public’s mindset begins to slacken and cooperation with epidemic prevention decreases, making it more difficult to carry out prevention and control work. The willingness of individuals to participate in preventive health measures depends on their level of knowledge [6]. Therefore, relevant government departments and the media can disseminate the knowledge of COVID-19 to the public by producing videos to improve the public’s knowledge, which can raise awareness and cooperation of the public in epidemic prevention, while it is true that increased coverage of health news is positively correlated with prevention behavior, this relationship only exists for those who regularly follow health news [7]. Furthermore, being exposed to the medium, there is a difference in the amount of knowledge individuals acquire from COVID-19 videos as the information is disseminated. Therefore, exploring the knowledge learning process of COVID-19 video viewers can help to understand how individuals acquire COVID-19 knowledge through COVID-19 videos. Only by understanding the knowledge learning process of individuals can we better use these videos to disseminate prevention knowledge and improve the cooperation of prevention.

In general, to understand the knowledge learning process of COVID-19 video viewers, this research constructs a knowledge learning path model of COVID-19 video viewers, which helps to analyze how viewers acquire knowledge from COVID-19 videos.

## 2. Literature Review

### 2.1. Cognitive Mediation Model (CMM)

**Figure 1:**
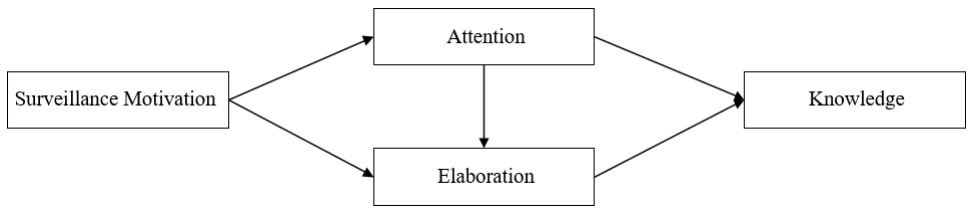
Cognitive Mediation Model.

The cognitive mediation model was proposed in the context of exploring the learning process of political news [8]. The model is widely used to understand the process by which the public learns about politics and engages with it. This model describes how individuals exposed to the media are motivated to process information and acquire knowledge from the media. It contains three elements, namely, surveillance motivation, information processing, and knowledge, among which information processing includes attention and elaboration. Surveillance motivation refers to individuals’ attitudes aimed at using media to stay informed about things. It is an important motivation for media engagement by motivating individuals to adopt information-processing behaviors [9]. Information processing consists of two components—attention and elaboration. Attention refers to the tendency of an individual to mentally focus on specific content in the media, which can result in selective exposure and perception of specific content. Elaboration refers to the tendency of individuals to make connections between the information they are exposed to and their past experiences and existing knowledge, and to update the memory stores through refined processing. Knowledge refers to the memory formed through the recognition and processing of factual information. The relations in the original CMM are shown in Figure 1.

A considerable number of studies have been conducted to validate and extend the cognitive mediation model in different domains. Most studies have focused on individuals’ cognitive processes of learning for various types of news, such as science news on social media [10], news about climate change [11,12], health news reports [13], and political news [9,14]. However, few studies have applied the cognitive mediation model to the knowledge learning from videos. Many studies have not only validated the original cognitive mediation model but also extended the model. King and their colleagues [15] compared the information utility model, the cognitive mediation model, and the integrated model to analyze which model could better explain the knowledge-learning mechanism of cancer news. In addition, some studies have extended the model to behavioral willingness and behavior. Ho [16] analyzed the factors influencing public knowledge acquisition and willingness to adopt preventive behaviors during the pandemic H1N1, while Jin and co-authors [17] analyzed the path from online health knowledge seeking to colorectal cancer screening based on health literacy and cognitive mediation model. In the health field, several scholars have added perceived risk to the model, Zhang and Yang [18] have substituted perceived risk and information seeking for surveillance motivation to explore the effect of perceived risk on breast cancer patients’ willingness to examination intention. Lee et al. [6] substituted perceived risk for surveillance motivation to analyze the mechanisms of learning breast cancer knowledge of Singaporean women. However, few studies have added perceived risk as an additional variable to the model to analyze the effect of perceived risk on surveillance motivation. In this paper, we add the perceived risk of COVID-19 variable to the original model to investigate the cognitive process of knowledge learning of COVID-19 video viewers with the help of videos.

### 2.2. Dual Coding Theory (DCT)

Dual coding theory, first proposed by Allan Paivio in 1971 [19], posits that an individual’s perception of new knowledge relies on two separate and interrelated cognitive systems: the nonverbal system and the verbal system [20]. The non-verbal system focuses on processing non-verbally encoded information, such as visual images and expression reflections. The verbal system specializes in processing linguistically encoded information, such as text, the voice of the teacher teaching, etc. In the process of information awareness and processing, the two systems have three different ways of processing information [21]. The first is the representational style in which the external perceptual system directly activates the encoding activity of the verbal or nonverbal system; the second is the joint style in which the internal action activity between the representational units within a certain encoding system; and the third is the mutual, one-to-many activation action between two encoding systems. These three types of information processing allow for a mutually independent and related relationship between the linguistic and nonlinguistic coding systems.

Dual coding theory is often used in the research of the education industry to analyze the impact of linguistically and non-linguistically coded information on students’ learning performance [19,22,23,24]. Kusumawati and Rachmawati’s [22] study shows that applying dual coding theory to storytelling methods can enhance children’s vocabulary learning and reading comprehension. Based on dual coding theory, Liu et al. [23] investigated how texts and images in computer-assisted learning affect students’ learning attitudes, learning performance, and teacher instruction. In addition, dual coding theory provides two perspectives for analyzing the effects of information on individuals: verbal and nonverbal information. Yang et al. [25] analyzed the effect of the textual format and imagery format of online restaurant reviews on review usefulness and review enjoyment. Most of the studies have analyzed static information with the help of dual coding theory but rarely analyzed dynamic information, such as the information in videos. In this paper, based on the dual coding theory, the information in the COVID-19 video is divided into linguistically coded information and non-linguistically coded information, in which the text and voiceover in the video are taken as linguistically coded information and the images and emoticons in the video are taken as non-linguistically coded information. The cognitive processing and knowledge acquisition of individuals for these two types of coded information were explored in this paper.

### 2.3. Knowledge Learning through Videos

As a visual information carrier, video is often applied to the field of e-education. Especially during the COVID-19 epidemic, online videos have become the main channel for students to conduct knowledge learning. The powerful narrative style and rich content presentation of videos can enhance the viewer’s understanding of the video content. When teaching content or training content is presented in the form of a video, the viewer finds the video useful for knowledge learning [26,27]. Different types of videos have various effects on individual knowledge learning. Compared to traditional videos, interactive videos are more effective in improving students’ learning performance and learning satisfaction [28]. The characteristics of the video itself can also affect individual learning, for example, videos with subtitles have greater vocabulary learning benefits than videos without subtitles [29]. Besides, the change in video playback speed significantly affects the cognitive load of individuals, and a medium cognitive load is required to achieve the highest learning effect [30]. To better integrate video into the educational field, many researchers have proposed learning systems that incorporate multiple mechanisms to improve students’ knowledge acquisition and teachers’ lecture efficiency [31,32,33].

There are differences in the knowledge acquired by different individuals through videos. Individuals with different prior knowledge have the same level of participation in video lectures, but those with high prior knowledge watch videos more frequently, have a more active attitude towards watching videos and perform better in learning [34]. Existing studies have more often analyzed the association between individuals’ video learning behaviors and learning performance based on their video viewing logs, such as analyzing the impact of video viewing frequency and viewing time [35,36] and user actions during video viewing [37] on individuals’ learning performance. Some studies have also analyzed the effect of video viewing on individuals’ motivation to learn knowledge, for example, cancer-related video presentations can increase motivation in cancer patients under conditions of high distress and disease threat [38]. However, fewer studies have analyzed the cognitive processes by which individuals learn knowledge through video. Especially during the COVID-19 epidemic, the COVID-19 video can help the public to reduce anxiety and improve knowledge [39]. Analyzing the knowledge-learning process of individuals in the COVID-19 videos can help improve the public’s perception of the epidemic. Therefore, this paper explores the knowledge-learning process of COVID-19 video viewers based on the cognitive mediation model.

## 3. Research Hypothesis and Model

### 3.1. Research Hypothesis

#### 3.1.1. Perceived Risk of COVID-19 and Surveillance Motivation

The health belief model considers perceived risk as a key determinant of individual engagement in preventive behavior [40]. Individuals are more likely to take precautionary measures and actions when they perceive the epidemic to be serious or a threat to themselves. In addition, an individual’s perceived risk can also influence emotional risk perceptions (e.g., negative emotions such as anxiety and fear). When individuals perceive COVID-19 as serious and risky, they will develop concern and fear about the disease. Such emotions motivate individuals to seek information about COVID-19 and to keep an eye on its development to meet the individual’s need to cope with the risk of COVID-19 and to appease negative emotions. The act of seeking COVID-19-related information and maintaining constant attention to COVID-19 developments motivates individuals to monitor the epidemic. Therefore, the following hypothesis is proposed:

**Hypothesis 1** **(H1).** *An individual’s perceived risk of COVID-19 positively influences their surveillance motivation to the epidemic.*

#### 3.1.2. Surveillance Motivation, Attention, and Elaboration

Surveillance motivation as an intention does not, in itself, directly produce cognitive and behavioral outcomes [41]. The cognitive mediation model suggests that individuals are motivated by surveillance motivation to pay attention to news and refine the information, and then become aware of what is happening in their environment and make decisions [42].

A comparative study has found the differences in the information processing of verbally encoded information, non-verbally encoded information and asynchronous encoded information. Besides, the level of cognitive growth of them is also varied [43,44,45]. To analyze the difference of the effects of various encoded information in the videos, this paper divides the information processing (attention and elaboration) into the processing of linguistically coded information and non-linguistically coded information. In the subsequent hypotheses, the two encoded information both are discussed.

When confronted with a huge amount of information, individuals selectively identify information based on personal preferences such as relevance to their needs, importance, and personal interests [46]. An individual’s willingness to make information choices is to satisfy information-seeking needs. Influenced by the motivation to stay informed about the pandemic, individuals might seek information about COVID-19 to understand its status and keep on going with the latest information to track its development. The need to access information about the pandemic leads individuals to pay more attention to information about COVID-19 on video platforms. Therefore, this paper proposes the following hypotheses:

**Hypothesis 2a** **(H2a).** *An individual’s surveillance motivation to COVID-19 is positively correlated with their attention to non-verbally encoded information about COVID-19.*

**Hypothesis 2b** **(H2b).** *An individual’s surveillance motivation to COVID-19 is positively correlated with their attention to verbally encoded information about COVID-19.*

The human brain relates relevant information in a complex network [47,48] and creates instant access to relevant information through associative indexing during information handling and processing [49]. The process of refining information is known as elaboration, which is the process of thinking about a problem and integrating the acquired information into existing knowledge structures [50]. Elaboration, a process of renewal of self-knowledge structures and internalization of knowledge, is also influenced by surveillance motivation. Because the brain of human is more likely to be driven by the motivation to work which is to absorb new knowledge and relate to existing knowledge. Therefore, the following hypotheses are postulated:

**Hypothesis 3a** **(H3a).** *An individual’s surveillance motivation to COVID-19 is positively related to their elaboration about non-verbally encoded information about COVID-19.*

**Hypothesis 3b** **(H3b).** *An individual’s surveillance motivation to COVID-19 is positively related to their elaboration about verbally encoded information about COVID-19.*

#### 3.1.3. Attention and Elaboration

The cognitive mediation model considers attention to information as a prerequisite for elaboration [9], the tendency to concentrate mentally on information is accompanied by a selective allocation of individual cognitive abilities. During the information processing, the individual must first notice specific information in the media to activate the processing of information. Information that is not noticed will be ignored and is not available for elaboration. Therefore, when acquiring information through video, the individual’s attention to the COVID-19 information increases, the individual’s ability which integrates the acquired COVID-19 information into the existing knowledge structure and generates deeper cognitive relevance might become stronger. Hence, this paper posits the following hypotheses:

**Hypothesis 4a** **(H4a).** *An individual’s attention to non-verbally encoded COVID-19 information positively influenced their elaboration about non-verbally encoded COVID-19 information.*

**Hypothesis 4b** **(H4b).** *An individual’s attention to verbally encoded COVID-19 information positively influenced their elaboration about verbally encoded COVID-19 information.*

#### 3.1.4. Attention, Elaboration, and COVID-19 Knowledge

Both attention and elaboration may, to some extent, lead to an increase in the amount of knowledge an individual has. Attention enables the individual to notice information in the media, allowing the individual to gain initial learning. Elaboration allows information to be matched to integrate existing knowledge, refining the brain’s complex network of information about COVID-19 and increasing the total amount of knowledge. In addition, when recalling COVID-19-related knowledge, better connections between knowledge lead to an increase in the speed and likelihood of successful information retrieval, and knowledge recall becomes more profound. Therefore, this paper hypothesizes the followings:

**Hypothesis 5a** **(H5a).** *An individual’s attention to non-verbally encoded COVID-19 information has a positive effect on their COVID-19 knowledge.*

**Hypothesis 5b** **(H5b).** *An individual’s attention to verbally encoded COVID-19 information has a positive effect on their COVID-19 knowledge.*

**Hypothesis 6a** **(H6a).** *An individual’s elaboration about non-verbally encoded COVID-19 information has a positive impact on their COVID-19 knowledge.*

**Hypothesis 6b** **(H6b).** *An individual’s elaboration about verbally encoded COVID-19 information has a positive impact on their COVID-19 knowledge.*

### 3.2. Research Model

Individuals’ learning of new knowledge on the Internet may be influenced by their abilities. Learning ability varies between groups, with differences in cognitive thinking across gender and age groups. The higher the education of a person, the more knowledge he or she has accumulated, which indicates their ability to learn is also strong, but there is less room for learning growth. COVID-19 Video uses video as the information carrier. However, the sources of this information become more complex. Individuals have to familiarize themselves with the medium first, which requires a certain amount of learning costs. People who use the Internet frequently, especially those who watch videos regularly, are more accustomed to this way of accessing information and need to pay less learning costs. Therefore, in this paper, gender, age, education, daily leisure time online, and online video viewing time per day are considered as control variables.

**Figure 2:**
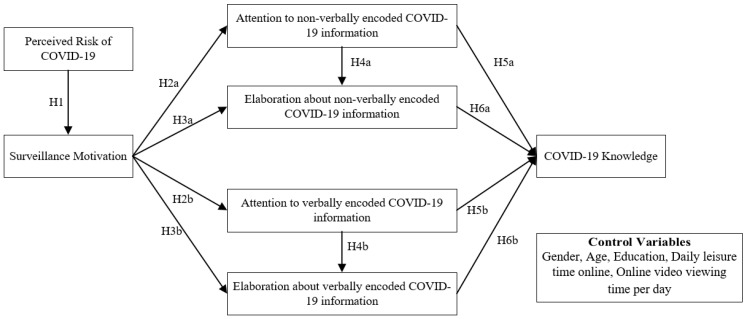
Knowledge learning path model of COVID-19 video viewers.

As shown in Figure 2, this paper proposes a knowledge learning path model of COVID-19 video viewers based on the cognitive mediation model and dual coding theory. In this paper, perceived risk of COVID-19 is incorporated into the model to explore the effect of perceived risk on surveillance motivation. The independent variable is an individual’s surveillance motivation to COVID-19, the mediating variable is an individual’s attention and elaboration about the content of the COVID-19 video, and the dependent variable is the COVID-19 knowledge acquired by the individual as a result of watching the video.

## 4. Materials and Methods

### 4.1. Variables and Measurement

In this paper, the online survey method was used to distribute questionnaires to viewers of the video platform. The design of the questionnaire includes screening items, demographic options, a video, and the main part. The screening items are used to screen out questionnaires with completed results that meet the requirements, including video viewing time, respondents’ subjective confirmation of complete viewing, and general knowledge question tests. The demographic options included gender, age, education, time spent online per day, and time spent watching videos per day.

The main part contained four latent variables, two manifest variables, and one measure of knowledge (Table 1). The latent variables include surveillance motivation (SM), elaboration about verbally encoded information (VE), elaboration about non-verbally encoded information (NE), and perceived risk of COVID-19 (PR), and the manifest variables include attention to verbally encoded information (VA), and attention to non-verbally encoded information (NA). For the study scenario of the COVID-19 video, this study considered that the verbally encoded COVID-19 information included the audio and text of the video, and the non-verbally encoded COVID-19 information included the image of the video. All latent and manifest variables were derived from existing literature, adapted in the context of this paper’s research scenario, and measured using a 5-point Likert scale. Eight judgment questions were designed according to the given COVID-19 video to measure the knowledge acquisition of individuals before and after watching the video. COVID-19 Knowledge (KN) was defined as the difference in the amount of knowledge before and after viewing the video.

### 4.2. Study Sample

This article is aimed at a group of people who have experience with video viewing. Video, as a form of media communication, has been integrated into the lives of most people. It can be said that almost everyone has watched videos. Take for example, one of the most popular video platforms in China. According to the third quarter of 2022, this platform had 90.3 million daily active users and 333 million monthly active users, both up 25% year-on-year. In this paper, questionnaires were randomly distributed to individuals with video viewing experience through the online questionnaire platforms. These platforms offer functions equal to Amazon Mechanical Turk. The questionnaires were distributed to groups that fit the research group of this paper, and the whole process was completely randomized.

### 4.3. Data Collection

302 questionnaires were collected from 16 October to 25 October 2022. To ensure the quality of the questionnaire, we excluded the questionnaires with short answer times, wrong answers to general knowledge trap questions, and non-compliant answers (same answer choices, obvious contradictions in logic). Finally, 255 valid questionnaires were obtained. Calderón-Mora et al.’s research showed that this sample size is representative of the population with video viewing experience and is consistent with the study population of this paper [53].

Table 2 shows the demographic results of the respondents. The percentage of male respondents was 41.96%, and the difference in the number of male and female respondents was not significant. In terms of age, the largest number of respondents were aged 18–25 at 50.98%, followed by 31–40 and 26–30 at 20% and 19.61% respectively. The results of education show that most of the respondents have a high level of education, with 18–40 respondents having a generally high level of education. Forty percent of respondents spend 2–4 h a day online, followed by 32.94% who spend 4–6 h a day online, and 80% of respondents use 1–3 h a day to watch online videos.

### 4.4. Methods

We used partial least squares structural equation modeling (PLS-SEM) to analyze the proposed research model, and the data analysis was partially done using SmartPLS 3.0 software. This is a powerful and very useful software for modeling partial least squares structural equations.

Firstly, we examined the reliability and validity of the constructs of the questionnaire, which were used to assess the stability and validity of the model. Reliability analysis is used to measure the consistency and stability of the results, and the measures include composite reliability (CR) and Cronbach’s α. Validity is used to test the validity of the model and includes discriminant validity and convergent validity.

Secondly, we tested the fitness of the model, which indicated the degree of fit of the sample data to the model. In this paper, we use SmartPLS 3.0 software to evaluate the fitness of the model. Model fitness metrics in SmartPLS include standardized root mean square residual (SRMR), normed fit index (NFI), d_ULS, and d_G [54].

Then, we validated the hypotheses using structural equation modeling, and the test method used was bootstrapping repeated sampling applicable to small samples, with the number of times set to 5000.

Finally, we tested the mediating effects of our model using SmartPLS 3.0 software. The test method we used is the new mediating effect test proposed by Wen and Ye [55].

## 5. Results

### 5.1. Reliability and Validity Tests

Reliability and validity testing are important indicators used to assess the quality of questionnaire data. Table 3 shows the reliability and convergent validity of the latent variables. Since VA and NA are manifest variables which are measured by one question, we did not put them in Table 3. Reliability analysis includes two metrics, composite reliability (CR) and Cronbach’s α. In general, questionnaires with Cronbach’s α and CR of 0.7 or above are satisfying the reliability requirement. Table 3 shows that both Cronbach’s α and CR exceed 0.7, indicating the favorable reliability of the questionnaire.

Validity includes discriminant validity and convergent validity. Convergent validity is measured by the average extracted variance (AVE), and the model is considered to have good convergent validity when the AVE is greater than 0.5. In Table 3, the AVE of each variable in the table is greater than 0.5, which meets the requirement.

Discriminant validity is used to measure the degree of discrimination between latent variables [56]. In Table 4, the diagonal values of the discriminant validity matrix are the square root of the AVE corresponding to each variable. As shown in Table 4, the square root of the AVE for each variable is greater than the correlation coefficient between the variables, indicating that the model in this study has good discriminant validity.

### 5.2. Model Fitness Assessment

The fitness of the model needs to be tested before the hypothesis test used structural equation modeling. In the SmartPLS3.0 software, there are several metrics, such as standardized root mean square residual (SRMR), normed fit index (NFI), d_ULS, and d_G [54]. SRMR, an absolute goodness-of-fit indices, is used to assess the average size of the difference between the observed and expected correlation matrices. According to Hu and Bentler [57], SRMR < 0.1 is acceptable, while a more stringent criterion is SRMR < 0.8. The SRMR value of our research model is 0.026, which meets the criteria. NFI, proposed by Bentler and Bonett [58], takes a value between 0 and 1, and the closer it is to 1, the better the model fit is. It is generally greater than 0.9 as the judgment criterion [54]. The NFI value of our model is 0.921, which fits the criteria. d_ULS and d_G, also known as the squared Euclidean distance and the geodesic distance, were proposed by Dijkstra and Henseler [59] as full adaptation criteria. It is required that the two values should be less than 0.95 [54]. The d_ULS and d_G of our model are 0.137 and 0.313, respectively, which meets the standard values. Through the above indicators, it can be concluded that the model proposed in this paper has good fitness and can be tested for structural model test.

### 5.3. Hypothesis Test

**Figure 3:**
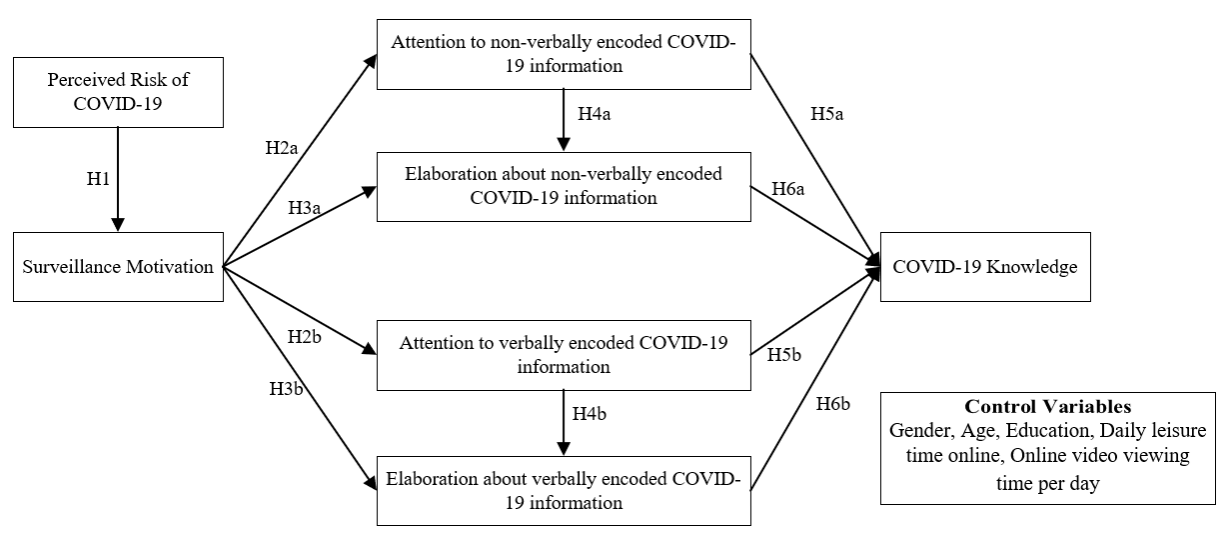
Knowledge learning path model of COVID-19 video viewers. * *p* < 0.05, ** *p* < 0.01, *** *p* < 0.001.

To test the hypothesis, this paper performed a structural model test used the SmartPLS3.0 software. The test method used was bootstrapping repeated sampling applicable to small samples, and the number of times was set to 5000. Before analyzing the results of hypothesis testing, this paper analyzed the effect of control variables on knowledge. The experimental results showed that among the control variables, only the length of video viewing per day had a significant positive effect on knowledge (β = 0.156, *p* = 0.037). That is, the longer the duration of video viewing per day, the more familiar the individual is with this medium of simultaneous verbal and nonverbal encoding and the better the ability to acquire knowledge. Based on the analysis of the structural equation test, this study maps the path analysis of knowledge learning of the COVID-19 video viewers (Figure 3). All the hypotheses proposed in this paper were supported and analyzed as follows.

#### 5.3.1. Perceived Risk of COVID-19, Surveillance Motivation, and Mediating Variables (Attention and Elaboration)

From Table 5, we can see that perceived risk of COVID-19 has a significant positive effect (β = 0.393, *p* < 0.001) on surveillance motivation. The hypotheses between surveillance motivation and information processing mediating variables were tested as follows. Surveillance motivation to COVID-19 has a positive effect on attention to non-verbally encoded COVID-19 information (β = 0.172, *p* < 0.001), elaboration about non-verbally encoded COVID-19 information (β = 0.274, *p* < 0.001), attention to verbally encoded COVID-19 information (β = 0.151, *p* = 0.003), and elaboration about verbally encoded COVID-19 information (β = 0.271, *p* < 0.001). Comparing the results, we can see an interesting point that surveillance motivation has a greater effect on elaboration than it does on attention. Maybe it’s because attention allows individuals to focus their minds selectively on the COVID-19 information, while elaboration further helps individuals to understand the COVID-19 information and enrich their knowledge structure about COVID-19. Out of the need to satisfy the surveillance motivation, elaboration goes a little deeper. In general, H1, H2a, H2b, H3a, and H3b are all supported.

We also can see in Table 5 that tests of the relationship between the mediating variables revealed a significant positive correlation between individuals’ attention and elaboration. Whether the information of videos is verbally encoded or non-verbally encoded, an individual’s attention is positively related to their elaboration. The influence coefficients for verbally and non-verbally encoded information are 0.498 and 0.369, respectively. Thus, H4a and H4b were supported.

#### 5.3.2. Mediating Variables (Attention and Elaboration) and Knowledge

As shown in Table 6, it can be seen that all mediating variables have a significant positive effect on knowledge. There are significant positive effects of NA (β = 0.144, *p* < 0.002) and NE (β = 0.154, *p* < 0.02), VA (β = 0.165, *p* < 0.009) and VE (β = 0.143, *p* < 0.044) on KN. Thus, supporting the proposed H5a, H5b, H6a, and H6b in this paper. In addition, we can also see that with the same information processing, the difference in the effect of information processing on knowledge between non-verbally coded information and verbally coded information method is not significant.

### 5.4. Mediating Effect Test

Since our research model is based on the cognitive mediation model, the mediating effect needs to be tested. In this paper, we used SmartPLS 3.0 software to test the mediating effect of our model. The test results are shown in Table 7. From Table 7, we can see that the total and mediating effects of all mediating paths are significant, which indicates that the mediating variables all play a good mediating role between SM and KN [55]. We can also see that the direct effects of all mediating paths are not significant, which suggests that there is only mediating effects existing in the model. That is, the surveillance motivation is acting on knowledge exclusively through attention and elaboration.

## 6. Discussion

### 6.1. Principal Findings

In terms of control variables. Based on the demographic results of the respondents, the study found that the majority of respondents were in the age range of 18–25, followed by 26–30 and 31–40. This is in line with the distribution structure of current Internet users, i.e., young people are the main force of the current Internet and are active users of the Internet [60]. In addition, the level of education of the respondents is generally higher, which is in line with the age distribution of the respondents. Individuals with more accumulated knowledge tend to place more importance on knowledge acquisition, and such groups are more willing to watch videos for learning [34]. Most of the respondents spend 1–3 h a day watching videos, and this paper found that the longer individuals watch videos, the more knowledge they acquire from them. This may be because the longer the video is watched, the more familiar the individual becomes with the use of this medium, and therefore the greater the ability to extract information from it. This shows that video has become an important channel for people to obtain information and an important vehicle for knowledge learning.

In terms of knowledge learning paths. The findings in this paper suggest that individuals who fear harm from COVID-19 and value the severity of the epidemic are more likely to want to maintain ongoing concern about the outbreak. This is because the stronger the perceived risk of COVID-19, the more individuals fear they will be harmed, and the better they can do in terms of understanding COVID-19 and taking preventive measures [18]. In addition, the relationship between surveillance motivation and knowledge is indeed mediated by attention and elaboration [20]. Individuals exposed to the COVID-19 videos, under the control of the surveillance motivation of staying informed about the epidemic, motivate themselves to pay attention to the information in the videos and to engage in elaboration such as thinking and knowledge integration. Both processes of information processing, attention and elaboration, can increase the knowledge of individuals from watching the COVID-19 video. Attention to the information in the video makes individuals aware of the information and make initial learning of the information, while elaboration about the COVID-19 information makes individuals deeply understand and remember the knowledge. Although previous studies have examined individuals’ knowledge learning paths based on cognitive mediation models, most of them focused on news knowledge learning [9,10,14]. In this paper, we analyzed the video knowledge learning paths of individuals based on the new scene of COVID-19 videos with the help of the cognitive mediation model and dual coding theory. Similar to previous research results, the video-based learning paths also verified the hypothesized relationships of the original cognitive mediation model, and the relationships between the variables were all significantly positive.

In terms of the level of effects, the degree of influence of COVID-19’s surveillance motivation on the COVID-19 video information elaboration is greater than its influence on the attention. Attention is the formation of initial cognition of information by creating personal awareness of COVID-19 information, and elaboration is the deep processing of COVID-19 video information [9]. Compared to attention, elaboration makes individuals remember the information more deeply and assigns personal understanding to the information, which is more satisfying for surveillance motivation. Numerous scholarly studies have also verified that surveillance motivation has a greater effect on elaboration than on attention [16,61]. In addition, there is little difference in the impacts on knowledge of verbally and non-verbally encoded information, which indicates that for the information in the videos, an individual’s ability to obtain information from sounds and words is close to that of information from images. This demonstrates that verbal and non-verbal information in videos are equally useful for individual knowledge learning. This is different from most previous studies. Farley and their colleagues suggested that words with visual images attached were found to be more beneficial to students’ word recall than words without images [62]. Similarly, Lee et al. argued that participants were able to recall more information and elaborate more ideas related to the information when science news content was presented in an infographic compared to text [63]. Most studies have demonstrated that images tend to facilitate individual knowledge absorption more than text. However, this paper finds that there is no significant difference between the text, voiceover, and images in videos for individual knowledge absorption. This is an interesting phenomenon, suggesting that when different verbally encoded information is blended in a video, there is no significant difference in individuals’ ability to learn the two types of information.

### 6.2. Theoretical Contributions

There are three theoretical contributions in this paper:

Firstly, incorporated the perceived risk variable into the cognitive mediation model. Several studies have been conducted to validate and extend the cognitive mediation model. However, each scholar’s perspective of model expansion is different. King and their colleagues [15] analyzed problems from the perspective of model integration by integrating the cognitive mediation model with the information utility model. Some scholars have also extended the knowledge dependent variable in the original model to the behavioral level by expanding the perspective horizontally backward [16,17]. Although some scholars have considered the application of perceived risk in cognitive mediation models before this paper [6,18]. However, fewer studies have put perceived risk as an additional variable in the model to extend the model horizontally forward, that is, to explore the effect of perceived risk on surveillance motivation. In this paper, we validated assumptions of the original model [9] and also the extended model with the addition of perceived risk variable.

Secondly, innovation in the application of dual coding theory. At present, the application of dual coding theory is mainly focused on the field of education, and most of the studies mainly focus on the influence of two types of linguistically coded information in teaching content on the teaching effect [19,22,23,24], but few studies on social media content for the general public. In the existing related studies, more static information is analyzed from dual coding theory [25], but rarely two types of linguistically encoded information in dynamic information such as videos are analyzed. In this paper, we applied the dual coding theory to the information classification of video content, and treat the voiceover and text in the video as verbally coded information, while the images that appear in the video without text are treated as non-verbally coded information.

Finally, the innovation of context. Studies have been conducted to investigate individual knowledge learning in terms of types of videos [28] and video browsing habits [35,36,37]. Most of the content focuses on the teaching and learning domain, and this part of the study focuses on the impact of video on teaching and learning outcomes, with the majority of the study population being students [28], health care professionals [39], and patients with specific diseases [38]. However, there are relatively few studies on the knowledge learning of the COVID-19 videos. In this paper, we studied the knowledge learning mechanism of COVID-19 video viewers, and the group analyzed is the Internet population with Internet access. By understanding the COVID-19 video knowledge learning mechanism of this group, it helps relevant departments to take measures to improve the knowledge and prevention awareness of this group.

### 6.3. Practical Contributions

This paper has three practical contributions.

Firstly, by analyzing the knowledge learning pathway of COVID-19 video viewers, this paper can help public health departments and media to understand the knowledge learning process of individuals exposed to COVID-19 information.

Secondly, we examined the components of video and revealed that verbally encoded and non-verbally encoded information in the video have the same status and importance. To increase the amount of learning of viewers, videos should have information embedded in both verbally encoded content such as voiceover and text, and non-verbally encoded content such as images. The video format that uses only irrelevant image coding such as expression packs, black screens, and portraits, and only linguistic coding such as invalid BGM, should be abandoned.

Finally, we also verified the positive effect of perceived risk of COVID-19 on surveillance motivation. The public health sector should correctly popularize and publicize the devastation and severity of COVID-19, especially for localized outbreaks. When localized outbreaks are serious, proper science popularization of the status and development of the epidemic can enable the public to correctly perceive the severity of COVID-19 and appropriately guide the public’s motivation to learn about COVID-19.

## 7. Limitations and Prospects

Firstly, the sample size obtained is small, and there may be problems of data bias, so the conclusions drawn may not apply to the study in general. Subsequent studies can verify and supplement the conclusions of this paper by expanding the sample size or other research methods. Second, because of the unobservable nature of the behavior, the model in this paper was not extended to public health behavior, and subsequent studies can expand based on the model to analyze the effect of the COVID-19 video on individual health behavior. Finally, due to the limitations of the experimental conditions, only one video was provided to the subjects for viewing, and the video content was fixed and equivalent, while in reality, individuals tend to acquire knowledge from multiple videos based on their habits. Later studies can provide a simulated environment based on users’ habits in Internet video content platforms for research.

## 8. Conclusions

Based on the cognitive mediation model and combined with dual coding theory, this paper proposes a knowledge learning path model of COVID-19 video viewers. It is found that the perceived risk of COVID-19 is positively correlated with surveillance motivation, and the more severe people perceive the risk of COVID-19, the greater the motivation to stay informed about COVID-19. In addition, attention and elaboration in information processing do play a mediating role in surveillance motivation and knowledge, suggesting that individuals with learning motivation need to go through the processing of video information—attention and elaboration—to acquire knowledge. The extended model proposed in this paper not only validates the hypothesized relationships of the original model, but also extends the application of the cognitive mediation model in the field of video knowledge learning. Unlike previous studies, this study not only expands the cognitive mediation model and dual coding theory, but also applies them to the new context of COVID-19 videos. The shortcomings of this study are the small sample size and the room for model expansion. Thus, future research can increase the sample size and improve the existing model.

## Figures and Tables

**Table 1:** Questionnaire Design.

Type	Variable	Factor	Question Items	Source
Latent Variable	Surveillance Motivation	SM1	I hope to know the current issues and events of COVID-19.	[9]
		SM2	I hope to know the future development of COVID-19.	
	Elaboration about verbally encoded information	VE1	I listened carefully to the dubbing of the video and read the text message carefully.	[42,46]
		VE2	When I hear the video’s voiceover and see the text, I think about it.	
		VE3	When I hear the video’s voiceover and see the text, I associate it with other known relevant information.	
	Elaboration about non-verbally encoded information	NE1	I carefully looked at the image information in the video.	
		NE2	When I see the image in the video, I do relevant thinking.	
		NE3	When I see the image in the video, I associate it with other known relevant information.	
	Perceived Risk of COVID-19	PR1	I am worried that I may infect with COVID-19.	[51]
		PR2	I’m worried about my family getting COVID-19.	
		PR3	I’m worried about COVID-19 happening in the area close to me.	
		PR4	I am concerned that COVID-19 will become a health issue.	
Manifest Variable	Attention to verbally encoded information	VA	When watching the video, I pay attention to the voiceover and text of the video.	[16,52]
	Attention to non-verbally encoded information	NA	When watching the video, I focus on the image of the video.	
COVID-19 Knowledge	1. New Coronavirus (2019-nCoV) Nucleic Acid Detection Kit uses the reverse transcription RPA method.			
	2. The new coronavirus is composed of GACT.			
	3. The RNA genetic material is detected in the New Coronavirus (2019-nCoV) Nucleic Acid Test.			
	4. When the new coronavirus “evidence of guilt” binds to the sequence in the fluorescent probe, a light bulb and a switch are connected, and with the help of the switch, the light bulb comes on.			
	5. In the amplification session, after 35 cycles, the initial nucleic acid sequence will be amplified to 34.3 billion copies.			
	6. In the amplification session, 55 degrees Celsius corresponds to the operation of synthesizing double-stranded DNA.			
	7. The results of the Nucleic Acid Test are available in approximately 4-6 h with no backlog of sample volume.			
	8. The fluorescent probe used in the New Coronavirus (2019-nCoV) Nucleic Acid Test is a small nucleotide sequence with a fluorescent group on one end and an inhibitory group on the other.			

**Table 2:** Demographic Results.

Variable	Value	Frequency	Percentage (%)
gender	male	107	41.96
	female	148	58.04
age	<18	2	0.78
	18–25	130	50.98
	26–30	50	19.61
	31–40	51	20
	41–50	8	3.14
	51–60	13	5.1
	>60	1	0.39
education	junior high school and below	3	1.18
	high school or technical secondary school	9	3.53
	university or junior college	186	72.94
	master or above	57	22.35
daily leisure time online	less than 2 h	16	6.27
	2–4 h	102	40
	4–6 h	84	32.94
	6–8 h	37	14.52
	more than 8 h	16	6.27
online video viewing time per day	less than 1 h	24	9.41
	1–2 h	127	49.8
	2–3 h	77	30.2
	3–4 h	15	5.88
	more than 4 h	12	4.71

**Table 3:** Reliability and convergent validity.

Variable	Factor	Factor Loadings	Cronbach’s α	CR	AVE
Surveillance Motivation (SM)	SM1	0.897	0.733	0.882	0.789
	SM2	0.879			
Elaboration about verbally encoded information (VE)	VE1	0.863	0.835	0.9	0.751
	VE2	0.876			
	VE3	0.86			
Elaboration about non-verbally encoded information (NE)	NE1	0.804	0.745	0.854	0.66
	NE2	0.834			
	NE3	0.799			
Perceived Risk of COVID-19 (PR)	PR1	0.762	0.776	0.855	0.595
	PR2	0.773			
	PR3	0.769			
	PR4	0.781			

**Table 4:** Discriminant Validity.

	SM	NE	VE	PR
SM	0.888			
NE	0.338	0.813		
VE	0.349	0.417	0.866	
PR	0.392	0.214	0.242	0.771

**Table 5:** Hypothesis testing of Perceived Risk of COVID-19, Surveillance Motivation, and Mediating variables.

Hypothesis	Path	Path Coefficients (β)	T-Statistic	Is the Hypothesis Supported
H1	PR -> SM	0.393 (***)	5.424	Yes
H2a	SM -> NA	0.172 (***)	2.762	Yes
H3a	SM -> NE	0.274 (***)	4.776	Yes
H2b	SM -> VA	0.151 (**)	2.144	Yes
H3b	SM -> VE	0.271 (***)	4.474	Yes
H4a	NA -> NE	0.369 (***)	5.211	Yes
H4b	VA -> VE	0.498 (***)	6.79	Yes

*** *p* < 0.001, ** *p* < 0.01.

**Table 6:** Hypothesis testing of mediating variables on COVID-19 knowledge.

Hypothesis	Path	Path Coefficients (β)	T-Statistic	Is the Hypothesis Supported
H5a	NA -> KN	0.144 (**)	2.339	Yes
H6a	NE -> KN	0.154 (*)	2.266	Yes
H5b	VA -> KN	0.165 (**)	2.452	Yes
H6b	VE -> KN	0.143 (*)	2.044	Yes

** *p* < 0.01, * *p* < 0.05.

**Table 7:** Results of the mediating effect test.

Mediating Path	Total Effects	Mediating Effects	Direct Effects	Test Result
SM-NA-KN	0.808 (***)	0.254 (***)	−0.041	complete mediation
SM-NE-KN	0.808 (***)	0.079 (**)	−0.041	complete mediation
SM-NA-NE-KN	0.808 (***)	0.143 (**)	−0.041	complete mediation
SM-VA-KN	0.808 (***)	0.172 (*)	−0.041	complete mediation
SM-VE-KN	0.808 (***)	0.066 (*)	−0.041	complete mediation
SM-VA-VE-KN	0.808 (***)	0.135 (*)	−0.041	complete mediation

*** *p* < 0.001, ** *p* < 0.01, * *p* < 0.05.

## Data Availability

Not applicable.

## Abbreviations

The following abbreviations are used in this manuscript:

SM	Surveillance Motivation
VE	Elaboration about verbally encoded information
NE	Elaboration about non-verbally encoded information
PR	Perceived Risk of COVID-19
VA	Attention to verbally encoded information
NA	Attention to non-verbally encoded information
KN	COVID-19 Knowledge
SRMR	Standardized Root Mean Square Residual
NFI	Normed Fit Index
d_ULS	the squared Euclidean distance
d_G	the geodesic distance
